# The Concordance Repertory of the More Characteristic Symptoms of the Materia Medica

**Published:** 1890-05

**Authors:** 


THE CONCORDANCE REPERTORY OF THE MORE CHARACTERISTIC SYMPTOMS OF THE MATERIA MEDICA. By William D. Gentry, M. D. Vol. I. New York; A. L. Chatterton & Co.

We are in receipt of the first volume of this laborious work. It contains mind and disposition, head and scalp, eyes, ears, nose, face.

For this work the author deserves the gratitude and praise of every follower of Hahnemann, not one of whom can afford to be without it. As the title implies, it is a concordance.

The work is dedicated "to all students of materia medica, and to all engaged in the practice of medicine according to the law of *Similia similibus curantur.*" To all of these we say that it is unique, and fulfils all the claims made for it, and if we were to fill pages with commendations we could say no more than that. Dr. Gentry will please accept our hearty thanks, and we assure him we shall look forward to the completion of the work. G. H. C.

